# Preoperative biliary stenting is associated with increased bacterial contamination and postoperative inflammation in pancreatic head resections

**DOI:** 10.1186/s12893-026-04167-6

**Published:** 2026-08-29

**Authors:** Sara Al-Madhi, Aristotelis Perrakis, Sara Acciuffi, Fuad Al-Qahom, Maximilian Dölling, Seong Jeong, Peter Lemmer, Maksym Boruta, Mohammad Othman Alhomsi, Mihailo Andric, Frank Meyer, Roland S. Croner, Mirhasan Rahimli

**Affiliations:** 1 Department of General, Visceral, Vascular and Transplant Surgery, University Hospital Magdeburg, Leipziger Str. 44, Magdeburg, 39120 Germany; 2 Department of Gastroenterology, Hepatology and Infectious Diseases, University Hospital Magdeburg, Leipziger Str. 44, Magdeburg, 39120 Germany

**Keywords:** Preoperative biliary drainage, Pancreatic cancer, Bacterial contamination, Wound complications, Pancreatoduodenectomy

## Abstract

**Background:**

The role of preoperative biliary drainage (PBD) in patients with resectable pancreatic head cancer remains controversial. Although traditionally used to reduce hyperbilirubinemia, increasing evidence suggests an association with higher postoperative morbidity. This study evaluated the impact of preoperative biliary stenting on microbial contamination, postoperative inflammatory response, and clinical outcomes.

**Methods:**

This retrospective single-center study included 102 patients with preoperative cholestasis and hyperbilirubinemia who underwent pancreatic head resection between March 2017 and January 2024. Patients were divided into a stent group (*n* = 66) and a no-stent group (*n* = 36). Intraoperative bile cultures, perioperative parameters, postoperative complications, and serial laboratory values were analyzed.

**Results:**

Patients who underwent preoperative stenting showed significantly higher rates of positive intraoperative bile cultures (75.8% vs. 5.6%, *p* < 0.001), longer operative times (360.4 vs. 319.8 min, *p* = 0.038), and prolonged time to surgery (51.2 vs. 23.8 days, *p* = 0.001). Among culture-positive patients, contamination was predominantly polymicrobial (80.8%, 140 isolates in total), with Enterobacterales (43.6%) and Enterococcus species (35.7%) as the most frequent isolates and Candida species detected in 19.2% of these patients. Postoperative CRP levels were significantly higher in the stent group on postoperative days 3 and 5. Abdominal wound dehiscence occurred exclusively in stented patients (12.1% vs. 0%, *p* = 0.048). Rates of other complications, ICU stay, and hospital length of stay were comparable between groups.

**Conclusion:**

Preoperative biliary stenting is associated with predominantly polymicrobial bacterial bile contamination, longer operative times, increased postoperative inflammatory response and wound-related complications, while pancreas-specific complications were not significantly increased. These findings support a selective rather than routine use of biliary drainage in patients with resectable pancreatic cancer, and suggest that the microbial spectrum, including the detection of Candida species in nearly one fifth of culture-positive patients, should be considered when planning perioperative antimicrobial strategies.

## Introduction

Perioperative management of patients with obstructive jaundice due to pancreatic or distal bile duct malignancies remains a topic of ongoing debate. Preoperative biliary drainage (PBD) using endoscopic stents has traditionally been applied to reduce bilirubin levels and improve liver function prior to major surgery.

In patients with resectable pancreatic ductal adenocarcinoma, symptoms occur in up to 90% of cases, with jaundice presenting in 40% and biliary obstruction requiring stenting in 41% [1]. However, increasing evidence challenges the routine use of PBD in all patients.

A landmark randomized controlled trial demonstrated that routine biliary drainage prior to pancreatoduodenectomy resulted in significantly higher complication rates compared to early surgery without drainage [2]. Even among patients with hyperbilirubinemia requiring preoperative drainage, endoscopic approaches conferred significantly higher perioperative complication rates and prolonged hospital stays compared to percutaneous drainage [3].

A meta-analysis revealed significantly higher rates of overall complications and wound infections in patients who received preoperative biliary stenting [4]. When preoperative biliary drainage is performed prior to pancreatoduodenectomy, self-expanding metal stents (SEMS) have been associated with lower rates of cholangitis and postoperative pancreatic fistula compared to plastic stents [5]. Furthermore, preoperative endoscopic stenting has been associated with significantly higher rates of bacterial bile contamination compared to surgical drainage (94% vs. 7%), with concordant bacteria identified in 83% of postoperative abdominal infections. Moreover, the overall complication burden was significantly increased in patients who underwent preoperative stenting [6].

Despite mounting evidence against routine preoperative drainage, stenting rates have paradoxically increased, with the majority of procedures performed prior to surgical consultation, resulting in unnecessarily prolonged time to definitive resection [7]. Moreover, there remains insufficient evidence to guide the selection of drainage modality in patients who require biliary decompression [8].

While the association between preoperative drainage and increased postoperative morbidity is thus well established, several aspects remain relevant for current practice. The composition of the biliary microbial spectrum determines the appropriate perioperative antimicrobial strategy, including whether standard prophylaxis provides adequate coverage, so that reporting it alongside clinical outcomes has direct practical relevance. A further consideration is that in stented patients the preoperative bilirubin value reflects the post-drainage state, so that baseline comparability of the two groups can only be assessed if pre-drainage values are available. Finally, comparing the complication pattern between stented and non-stented patients can indicate whether contamination-related mechanisms or general surgical complexity predominate. Our study therefore aims to characterize the microbiological spectrum of biliary contamination, the perioperative inflammatory response, and the pattern of postoperative complications associated with preoperative biliary stenting in a contemporary Western cohort undergoing pancreatic head resection.

## Patients and methods

**Fig. 1:**
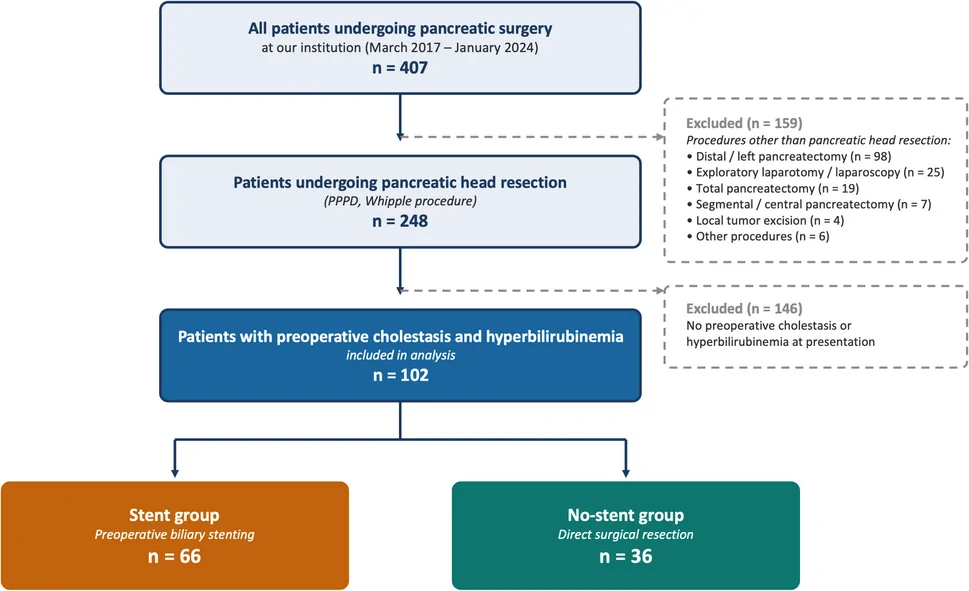
Flowchart of patient selection

This retrospective study included patients who underwent pancreatic head resection (either Whipple or pylorus-preserving pancreatoduodenectomy) for suspected pancreatic head carcinoma or distal cholangiocarcinoma with concomitant cholestasis and hyperbilirubinemia between March 2017 and January 2024. Patients were divided into two groups based on whether they received a preoperative biliary stent (Stent group, *n* = 66) or not (No-stent group, *n* = 36). A flowchart of patient selection is shown in Fig. 1. The indication for preoperative biliary stenting was determined individually in an interdisciplinary setting between visceral surgery and gastroenterology, without applying a fixed bilirubin cut-off. Factors favoring stenting included markedly elevated or dynamically worsening bilirubin, severe coagulopathy, cholangitis, relevant comorbidities, reduced performance status, and anticipated delay to surgery; patients without these features and scheduled for prompt resection proceeded directly to surgery.

Baseline characteristics such as age, sex, body mass index (BMI), ECOG performance status, diabetes mellitus, and smoking history were recorded. Preoperative laboratory values included CA 19 − 9, CRP, liver enzymes, cholestatic markers, coagulation parameters, and serum albumin. Perioperative parameters such as operative time, intraoperative blood loss, erythrocyte transfusion, ICU admission, ICU length of stay, total hospital length of stay (LOS), and time to surgery were collected. Time to surgery was defined as the time from diagnosis to surgery in the no-stent group, and from stent placement to surgery in the stent group. Intraoperative bile duct cultures were routinely obtained and evaluated microbiologically. All patients received standardized perioperative antibiotic prophylaxis at induction of anaesthesia according to our institutional protocol, irrespective of stent status. Preoperative and postoperative systemic antibiotic therapy beyond standard prophylaxis was recorded.

Postoperative complications were recorded and classified using the Clavien–Dindo grading system. Specific complications assessed included postoperative pancreatic fistula (POPF), bilio-digestive insufficiency, delayed gastric emptying (DGE), hemorrhage, intra-abdominal fluid collections, systemic inflammatory response syndrome (SIRS), and postoperative cholangitis. Routine laboratory markers were assessed preoperatively, on postoperative days 3 and 5, and at discharge.

### Statistical analysis

Continuous variables were expressed as means and standard deviations and compared using t-test. Categorical variables were compared using the Chi-squared or Fisher’s exact test, as appropriate. A p-value < 0.05 was considered statistically significant. All statistical analyses were performed using IBM SPSS Statistics, Version 26.0 (IBM Corp., Armonk, NY, USA).

## Results

### Patient characteristics

Baseline patient characteristics are summarized in Table 1. A total of 102 patients were included in the final analysis (Stent group: *n* = 66; No-stent group: *n* = 36). Both groups were comparable in terms of baseline characteristics. Age (69.6 ± 12.0 vs. 69.8 ± 11.1 years, *p* = 0.910), BMI (26.2 ± 4.9 vs. 25.4 ± 4.3, *p* = 0.416), and sex distribution (male: 53.0% vs. 52.8%, *p* = 0.981) showed no significant differences. ASA physical status was comparable between groups (ASA I/II/III/IV: 6.1%/40.9%/50.0%/3.0% vs. 5.6%/41.7%/47.2%/5.6%, *p* = 0.934). ECOG performance status, preoperative diabetes (31.8% vs. 36.1%, *p* = 0.660), and smoking history (27.3% vs. 25.0%, *p* = 0.804) were similarly distributed between groups. Neoadjuvant chemotherapy was administered in 4 of 102 patients (3.9%), all in the stent group (4/66, 6.1% vs. 0/36, 0%; *p* = 0.294).

**Table 1:** Patient characteristics

Parameter		Stent Group	No-Stent Group	*P*-value
Number of patients		66	36	
Gender	*male*	35 (53.0%)	19 (52.8%)	0.981
	*female*	31 (47.0%)	17 (47.2%)	
Age (years)		69.6 (12.0)	69.8 (11.1)	0.910
BMI		26.2 (4.9)	25.4 (4.3)	0.416
ASA	*ASA I*	4 (6.1%)	2 (5.6%)	0.934
	*ASA II*	27 (40.9%)	15 (41.7%)	
	*ASA III*	33 (50.0%)	17 (47.2%)	
	*ASA IV*	2 (3.0%)	2 (5.6%)	
Performance Status	*ECOG 0*	20 (30.3%)	16 (44.4%)	0.164
	*ECOG 1*	35 (53.0%)	18 (50.0%)	
	*ECOG 2*	11 (16.7%)	2 (5.6%)	
Preoperative Diabetes		21 (31.8%)	13 (36.1%)	0.660
Smoking history		18 (27.3%)	9 (25.0%)	0.804
Neoadjuvant chemotherapy		4 (6.1%)	0 (0.0%)	0.294

*ASA*  American Society of Anesthesiologists physical status classification, *BMI*  Body Mass Index

### Preoperative laboratory parameters

Pre-stenting bilirubin values were available for 51 of 66 stented patients and were comparable to the preoperative bilirubin levels of the no-stent group (212.9 ± 102.2 µmol/L vs. 199.9 ± 119.6 µmol/L, *p* = 0.586), as was the proportion of patients with severe hyperbilirubinemia ≥ 250 µmol/L (37.3% vs. 25.0%, *p* = 0.228).

Patients in the no-stent group exhibited significantly higher levels of liver enzymes and bilirubin compared to the stent group. Preoperative laboratory values are detailed in Table 2. Preoperative bilirubin was markedly elevated in the no-stent group (199.9 ± 119.6 vs. 79.3 ± 93.7 µmol/L, *p* < 0.001). Similarly, ALAT (3.0 ± 2.5 vs. 1.5 ± 1.9 µmol/s·L, *p* = 0.004), ASAT (2.4 ± 1.6 vs. 1.3 ± 2.5 µmol/s·L, *p* = 0.038), and GGT (13.1 ± 10.7 vs. 5.6 ± 4.7 µmol/s·L, *p* = 0.002) were significantly higher in the no-stent group. The Quick value was significantly higher in the stent group (87.7 ± 17.4 vs. 78.8 ± 16.4%, *p* = 0.014). Other parameters including CA 19 − 9, CRP, GLDH, PTT, albumin, and lipase showed no significant differences between groups.

**Table 2:** Preoperative laboratory values

Laboratory Parameter	Stent Group	No-Stent Group	*P*-value
*CA 19 − 9 (U/ml)*	906.3 (2208.4)	525.3 (813.4)	0.342
*CRP (mg/L)*	20.1 (29.4)	27.4 (42.8)	0.316
*ALAT (µmol/s·L)*	1.5 (1.9)	3.0 (2.5)	**0.004**
*ASAT (µmol/s·L)*	1.3 (2.5)	2.4 (1.6)	**0.038**
*GGT (µmol/s·L)*	5.6 (4.7)	13.1 (10.7)	**0.002**
*GLDH (nmol/s·L)*	642.4 (2548.2)	632.1 (731.1)	0.985
*Quick (%)*	87.7 (17.4)	78.8 (16.4)	**0.014**
*PTT (sec)*	32.9 (31.5)	30.5 (4.3)	0.648
*Albumin (g/L)*	36.9 (6.9)	36.8 (7.1)	0.933
*Bilirubin (µmol/L)*	79.3 (93.7)	199.9 (119.6)	**< 0.001**
*Lipase (µmol/s·L)*	5.0 (19.9)	4.3 (11.0)	0.855

*Albumin*  Serum albumin, *ALAT*  Alanine aminotransferase, *ASAT*  Aspartate aminotransferase, *Bilirubin*  Total bilirubin, *CRP*  C-reactive protein, *GLDH*  Glutamate dehydrogenase, *GGT*  Gamma-glutamyltransferase, *Lipase*  Serum lipase, *PTT*  Partial thromboplastin time, *Quick*  Prothrombin time (Quick value)

### Perioperative parameters and microbial contamination

Perioperative parameters are presented in Table 3. Time to surgery was significantly longer in the stent group (51.2 ± 62.4 vs. 23.8 ± 14.7 days, *p* = 0.001). The stent group showed significantly longer operative times (360.4 ± 101.7 vs. 319.8 ± 75.4 min, *p* = 0.038). Intraoperative blood loss (714.2 ± 848.2 vs. 489.0 ± 296.8 ml, *p* = 0.127) and transfusion requirements (22.7% vs. 22.2%, *p* = 0.953) were comparable between groups. Portal vein resection was performed in 6 of 66 stented patients (9.1%) and in 1 of 36 non-stented patients (2.8%), without reaching statistical significance (*p* = 0.416). ICU admission rates (95.5% vs. 86.1%, *p* = 0.196), ICU stay duration (4.5 ± 6.5 vs. 5.0 ± 7.0 days, *p* = 0.718), and overall hospital length of stay (25.4 ± 17.9 vs. 28.2 ± 13.0 days, *p* = 0.403) did not differ significantly.

**Table 3:** Perioperative parameters

Parameter	Stent Group	No-Stent Group	*P*-value
Number of patients	66	36	
Time to surgery (days)	51.2 (62.4)	23.8 (14.7)	**0.001**
Operation time (min)	360.4 (101.7)	319.8 (75.4)	**0.038**
Intraoperative blood loss (ml)	714.2 (848.2)	489.0 (296.8)	0.127
Intraoperative transfusion	15 (22.7%)	8 (22.2%)	0.953
Intraoperative transfusion (ml)	165.2 (416.8)	125.0 (241.9)	0.597
Portal vein resection	6 (9.1)	1 (2.8)	0.416
ICU admission	63 (95.5%)	31 (86.1%)	0.196
ICU stay duration (days)	4.5 (6.5)	5.0 (7.0)	0.718
LOS (days)	25.4 (17.9)	28.2 (13.0)	0.403
Positive intraoperative bile culture	50 (75.8%)	2 (5.6%)	**< 0.001**
Preoperative antibiotic therapy	22 (33.3%)	5 (13.9%)	**0.033**
Postoperative antibiotic therapy	29 (43.9%)	18 (50.0%)	0.557

*ICU*  Intensive Care Unit, *LOS*  Length of Overall Hospital Stay

Most notably, a significantly higher rate of positive intraoperative bile cultures was observed in the stent group (75.8% vs. 5.6%, *p* < 0.001), demonstrating substantial microbial contamination associated with preoperative stenting.

Preoperative systemic antibiotic therapy beyond standard prophylaxis was more frequent in the stent group than in the no-stent group (33.3% vs. 13.9%, *p* = 0.033), whereas postoperative antibiotic therapy was comparable between groups (43.9% vs. 50.0%, *p* = 0.557).

Post-procedural complications related to endoscopic stent placement were documented in 21 of 66 stented patients (31.8%), including post-ERC cholangitis (*n* = 16, 24.2%) and post-ERC pancreatitis (*n* = 4, 6.1%).

### Microbial spectrum of positive bile cultures

In the 52 patients with positive intraoperative bile cultures, a total of 140 microbial isolates were identified. Contamination was predominantly polymicrobial, with 80.8% of positive cultures yielding ≥ 2 organisms (mean 2.7 organisms per culture). The detailed organism distribution is shown in Table 4. Enterobacterales were the most frequently isolated group (43.6% of isolates), followed by Enterococcus species (35.7%, including one isolate of vancomycin-resistant E. faecium), Candida species (7.9%), Streptococcus species (5.7%), Staphylococcus species (2.9%), anaerobes (2.1%), and Pseudomonas aeruginosa (2.1%). Candida species were detected in 19.2% of culture-positive patients.

**Table 4:** Microbial spectrum of positive intraoperative bile cultures (*n* = 52 patients, 140 isolates)

Parameter	Value
*Patients with positive bile culture*	52 (51.0%)
*Total isolates*	140
*Mean organisms per positive culture*	2.7
*Monomicrobial cultures*	10 (19.2%)
*Polymicrobial cultures (≥ 2 organisms)*	42 (80.8%)

**Organism group**	**n (%) of isolates**
Enterobacterales*	61 (43.6%)
Enterococcus species (incl. 1 vancomycin-resistant E. faecium)	50 (35.7%)
Candida / yeast species†	11 (7.9%)
Streptococcus species	8 (5.7%)
Staphylococcus species	4 (2.9%)
Anaerobes**	3 (2.1%)
Pseudomonas aeruginosa	3 (2.1%)

*E. coli, Klebsiella spp., Enterobacter cloacae complex, Citrobacter spp., Proteus spp., Morganella morganii, Serratia spp., Hafnia alvei

**Bacteroides spp., Prevotella spp., Lactobacillus spp.

†Detected in 10/52 culture-positive patients (19.2%)

### Postoperative complications

Postoperative complications are summarized in Table 5. The incidence of postoperative pancreatic fistula (POPF) was similar between groups (13.6% vs. 16.7%, *p* = 0.680). No significant differences were observed for delayed gastric emptying (3.0% vs. 8.3%, *p* = 0.342), hemorrhage (7.6% vs. 8.3%, *p* = 1.000), bilio-digestive insufficiency (7.6% vs. 2.8%, *p* = 0.420), lymphatic fistula (21.2% vs. 22.2%, *p* = 0.906), or intra-abdominal fluid collections (9.1% vs. 13.9%, *p* = 0.512).

**Table 5:** Postoperative complications

Complication	Stent Group	No-Stent Group	*P*-value
*Number of patients*	66	36	
*POPF*	9 (13.6%)	6 (16.7%)	0.680
*Delayed gastric emptying*	2 (3.0%)	3 (8.3%)	0.342
*Hemorrhage*	5 (7.6%)	3 (8.3%)	1.000
*Bilio-digestive insufficiency*	5 (7.6%)	1 (2.8%)	0.420
*Lymphatic fistula*	14 (21.2%)	8 (22.2%)	0.906
*Intra-abdominal fluid collection*	6 (9.1%)	5 (13.9%)	0.512
*Wound infection*	8 (12.1%)	2 (5.6%)	0.488
*Abdominal wound dehiscence*	8 (12.1%)	0 (0.0%)	**0.048**
*Pulmonary embolism*	0 (0.0%)	1 (2.8%)	0.353
*Pleural effusion*	5 (7.6%)	6 (16.7%)	0.189
*Pneumonia*	4 (6.1%)	3 (8.3%)	0.695
*SIRS*	10 (15.2%)	2 (5.6%)	0.206
*Sepsis*	6 (9.1%)	1 (2.8%)	0.416
*Thrombosis*	10 (15.2%)	3 (8.3%)	0.373
*Myocardial infarction*	1 (1.5%)	1 (2.8%)	1.000
*UTI*	9 (13.6%)	6 (16.7%)	0.680
*New-onset diabetes mellitus*	1 (1.5%)	1 (2.8%)	1.000
*Renal insufficiency*	9 (13.6%)	4 (11.1%)	1.000
*Postoperative cholangitis*	2 (3.0%)	2 (5.6%)	0.612

*POPF*  Postoperative pancreatic fistula, *SIRS*  Systemic inflammatory response syndrome, *UTI*  Urinary tract infection

Wound-related complications showed notable differences. While wound infection rates were not significantly different (12.1% vs. 5.6%, *p* = 0.488), abdominal wound dehiscence occurred exclusively in the stent group (12.1% vs. 0%, *p* = 0.048).

Inflammatory and infection-related complications such as SIRS (15.2% vs. 5.6%, *p* = 0.206) and sepsis (9.1% vs. 2.8%, *p* = 0.416) showed numerically higher rates in the stent group without reaching statistical significance. Pulmonary complications such as pleural effusion (7.6% vs. 16.7%, *p* = 0.189) and pneumonia (6.1% vs. 8.3%, *p* = 0.695) were comparable.

Other complications including thrombosis, myocardial infarction, urinary tract infection, renal insufficiency, and postoperative cholangitis showed no significant differences between groups.

### Postoperative laboratory dynamics

Postoperative laboratory values revealed distinct patterns between groups. These are presented in Table 6. On postoperative day 3, CRP levels were significantly higher in the stent group (170.8 ± 93.8 vs. 107.1 ± 78.6 mg/L, *p* < 0.001), while GGT (2.5 ± 2.6 vs. 6.4 ± 4.9 µmol/s·L, *p* = 0.003) and bilirubin (24.5 ± 33.2 vs. 108.6 ± 69.4 µmol/L, *p* < 0.001) remained significantly higher in the no-stent group. This pattern persisted on postoperative day 5, with elevated CRP in the stent group (109.6 ± 84.5 vs. 76.3 ± 63.0 mg/L, *p* = 0.046) and persistently higher GGT (2.7 ± 2.0 vs. 5.8 ± 3.7 µmol/s·L, *p* < 0.001) and bilirubin (21.7 ± 26.2 vs. 75.5 ± 62.2 µmol/L, *p* < 0.001) in the no-stent group.

**Table 6:** Postoperative laboratory values

Laboratory Parameter	Stent Group	No-Stent Group	*P*-value
Laboratory Values on Postoperative Day 3			
* CRP (mg/L)*	170.8 (93.8)	107.1 (78.6)	**< 0.001**
* ALAT (µmol/s·L)*	2.9 (9.0)	2.2 (2.9)	0.677
* ASAT (µmol/s·L)*	4.2 (15.6)	2.0 (2.6)	0.478
* GGT (µmol/s·L)*	2.5 (2.6)	6.4 (4.9)	**0.003**
* GLDH (nmol/s·L)*	3756.6 (12086.9)	475.2 (1241.5)	0.190
* Albumin (g/L)*	25.2 (3.3)	26.0 (4.1)	0.446
* Bilirubin (µmol/L)*	24.5 (33.2)	108.6 (69.4)	**< 0.001**
* Lipase (µmol/s·L)*	0.5 (0.7)	0.3 (0.4)	0.440
* Quick (%)*	75.7 (14.4)	77.3 (20.7)	0.654
* PTT (sec)*	31.2 (6.2)	31.9 (5.8)	0.573
Laboratory Values on Postoperative Day 5			
* CRP (mg/L)*	109.6 (84.5)	76.3 (63.0)	**0.046**
* ALAT (µmol/s·L)*	2.1 (7.7)	2.0 (3.0)	0.928
* ASAT (µmol/s·L)*	2.8 (14.2)	1.7 (2.2)	0.647
* GGT (µmol/s·L)*	2.7 (2.0)	5.8 (3.7)	**< 0.001**
* GLDH (nmol/s·L)*	2495.3 (10644.8)	515.5 (1264.6)	0.391
* Albumin (g/L)*	25.1 (4.1)	25.0 (4.4)	0.975
* Bilirubin (µmol/L)*	21.7 (26.2)	75.5 (62.2)	**< 0.001**
* Lipase (µmol/s·L)*	0.2 (0.1)	0.4 (0.4)	0.079
* Quick (%)*	76.7 (15.8)	78.7 (18.7)	0.575
* PTT (sec)*	32.6 (12.3)	30.3 (9.9)	0.343
Laboratory Values at Time of Discharge			
* CRP (mg/L)*	44.5 (53.8)	43.6 (48.9)	0.935
* ALAT (µmol/s·L)*	2.8 (10.6)	1.2 (1.8)	0.412
* ASAT (µmol/s·L)*	9.7 (41.4)	0.7 (0.4)	0.124
* GGT (µmol/s·L)*	2.5 (1.8)	4.4 (5.3)	0.092
* GLDH (nmol/s·L)*	5245.4 (27756.2)	89.7 (62.0)	0.368
* Bilirubin (µmol/L)*	14.4 (16.7)	35.4 (30.3)	**0.001**

*Albumin * Serum albumin, *ALAT*  Alanine aminotransferase, *ASAT*  Aspartate aminotransferase, *Bilirubin*  Total bilirubin, *CRP*  C-reactive protein, *GLDH*  Glutamate dehydrogenase, *GGT*  Gamma-glutamyltransferase, *Lipase*  Serum lipase, *PTT*  Partial thromboplastin time, *Quick*  Prothrombin time (Quick value)

At the time of discharge, CRP levels had normalized in both groups (44.5 ± 53.8 vs. 43.6 ± 48.9 mg/L, *p* = 0.935). Bilirubin remained significantly higher in the no-stent group (14.4 ± 16.7 vs. 35.4 ± 30.3 µmol/L, *p* = 0.001), though substantially reduced from preoperative values. Other liver enzymes and coagulation parameters showed no significant differences at discharge.

### Pathological tumor characteristics

The pathological tumor characteristics, including pT stage, pN stage and tumor size, were comparable between groups (Table 7). pT distribution did not differ significantly between the stent and no-stent group (*p* = 0.090), nor did pN distribution (*p* = 0.277) or pathological tumor size (34.6 ± 15.1 vs. 36.9 ± 15.7 mm, *p* = 0.529).

**Table 7:** Pathological tumor characteristics

Parameter	Stent Group (*n* = 66)	No-Stent Group (*n* = 36)	*P*-value
*pT stage*			0.090
* pT1*	5 (8.3%)	1 (3.0%)	
* pT2*	21 (35.0%)	20 (60.6%)	
* pT3*	32 (53.3%)	12 (36.4%)	
* pT4*	2 (3.3%)	0 (0%)	
*pN status*			0.277
* pN0*	13 (21.7%)	8 (23.5%)	
* pN1*	18 (30.0%)	15 (44.1%)	
* pN2*	29 (48.3%)	11 (32.4%)	
*Tumor size (mm)*,* mean ± SD*	34.6 ± 15.1	36.9 ± 15.7	0.529

Percentages refer to patients with available staging data (pT: stent *n* = 60, no-stent *n* = 33; pN: stent *n* = 60, no-stent *n* = 34; size: stent *n* = 52, no-stent *n* = 29)

## Discussion

This retrospective comparative study demonstrates that preoperative biliary stenting in patients undergoing pancreatic head resection is associated with substantially higher rates of bacterial bile contamination and a distinct postoperative inflammatory response pattern. While preoperative stenting effectively reduced bilirubin levels and improved coagulation parameters prior to surgery, it was accompanied by a marked increase in positive intraoperative bile cultures compared to patients who proceeded directly to surgery. Beyond confirming the known association between stenting and bacterial contamination, our study characterizes the composition of this contamination in detail, demonstrates comparable baseline biliary obstruction in both groups on the basis of pre-stenting bilirubin values, and identifies a dissociated complication pattern in which wound-related but not pancreas-specific complications were increased.

### Microbial contamination and clinical implications

The most striking finding of our study was the markedly elevated rate of positive bile cultures in the stent group (75.8% vs. 5.6%, *p* < 0.001). This observation is consistent with previous reports demonstrating bacterial colonization rates exceeding 90% following endoscopic stenting [6]. The mechanism underlying this contamination is multifactorial, involving disruption of the sphincter of Oddi, creation of a foreign body nidus for biofilm formation, and potential introduction of bacteria during the endoscopic procedure itself.

Recent metagenomic analysis of biliary stents has revealed that 17 of the 36 prevalent bacterial species are common oral microbiome members that harbor dozens of biofilm-formation and antimicrobial resistance genes [9]. Furthermore, preoperative biliary drainage has been shown to shift the biliary microbiological spectrum toward polymicrobial colonization with a higher proportion of resistant microorganisms [10].

In our cohort, the microbial spectrum was predominantly polymicrobial (80.8% of positive cultures with ≥ 2 organisms, mean 2.7 organisms per culture), with Enterobacterales (43.6%) and Enterococcus species (35.7%) as the most frequent isolates. Of particular clinical relevance is the detection of Candida species in 19.2% of culture-positive patients, since the standard perioperative antibiotic prophylaxis does not provide antifungal coverage. The isolation of vancomycin-resistant E. faecium in one patient further illustrates the potential for clinically relevant resistance patterns in this population. Together, these findings suggest that in stented patients with positive intraoperative bile cultures, broadening of the empirical perioperative regimen – including consideration of antifungal coverage in selected cases – may be warranted, and that targeted culture-directed therapy should be initiated whenever feasible.

Importantly, prior research has demonstrated that bacteria cultured from bile in stented patients frequently match those subsequently identified in postoperative abdominal infections, with concordance rates reaching 83% [6]. This suggests that preoperative bile contamination may directly contribute to postoperative septic complications rather than representing an incidental finding. In our cohort, while individual infectious complications did not reach statistical significance, there was a consistent numerical trend toward higher rates of infection-related complications such as sepsis (9.1% vs. 2.8%) and wound infection (12.1% vs. 5.6%) in the stented group, accompanied by significantly elevated CRP on postoperative days 3 and 5, supporting this mechanistic hypothesis. Moreover, a recent study has demonstrated that positive intraoperative bile cultures combined with antibiotic resistance significantly increase the risk of postoperative pancreatic fistula [11].

The significantly higher incidence of abdominal wound dehiscence exclusively in the stent group (12.1% vs. 0%, *p* = 0.048) may represent a clinically relevant manifestation of this increased microbial burden. This finding is consistent with recent meta-analytic evidence including 39 studies involving over 33,000 patients demonstrating that preoperative biliary drainage significantly increases the risk of surgical site infections (risk ratio 1.84) [12]. Bacterial contamination of the operative field, particularly with bile spillage during pancreaticoduodenectomy, could impair wound healing and increase the risk of fascial dehiscence. This finding warrants particular attention to surgical technique and potentially more aggressive wound management strategies in patients with preoperative stents.

### Postoperative inflammatory response

Our analysis of postoperative laboratory dynamics revealed a distinctive pattern that merits consideration. Patients in the stent group exhibited significantly elevated CRP levels on postoperative days 3 and 5, despite lower preoperative bilirubin levels. This heightened inflammatory response may reflect the consequences of bacterial contamination, subclinical infections, or an exaggerated immune response to microbial products released during surgical manipulation of colonized bile ducts.

Conversely, the no-stent group maintained persistently elevated bilirubin levels throughout the postoperative period, reflecting the ongoing cholestasis that prompted consideration of preoperative drainage in clinical practice. However, despite higher bilirubin levels, these patients demonstrated lower inflammatory markers and comparable clinical outcomes, challenging the traditional paradigm that hyperbilirubinemia per se necessitates preoperative correction.

The normalization of CRP by hospital discharge in both groups, alongside the substantial reduction in bilirubin levels in the no-stent group, suggests that uncomplicated pancreaticoduodenectomy effectively resolves biliary obstruction without the need for preoperative intervention in many cases. This observation supports the findings of the landmark randomized trial demonstrating superior outcomes with early surgery compared to routine preoperative drainage [2].

### Perioperative outcomes and resource utilization

The significantly longer operative time in the stent group (360.4 vs. 319.8 min, *p* = 0.038) likely reflects technical challenges associated with inflammation and tissue edema resulting from stent placement. A randomized controlled trial comparing endoscopic and percutaneous biliary drainage demonstrated that endoscopic stenting resulted in significantly more difficult dissections, increased blood loss, and prolonged operative times in patients with resectable pancreatic head cancer [3]. While this 40-minute difference may seem modest, it translates to meaningful increases in anesthesia time, operating room costs, and potential surgeon fatigue during complex procedures.

The prolonged operative time and time to surgery observed in the stent group cannot be attributed to differences in tumor burden, since pT, pN, and pathological tumor size were comparable between groups (Table 7). Other contributing factors are more plausible, including local inflammation and tissue edema secondary to stent placement and post-procedural events, particularly cholangitis (24.2%) and pancreatitis (6.1%) in stented patients.

Despite the microbial burden and prolonged operative times, we did not observe significant differences in major surgical complications such as postoperative pancreatic fistula (POPF), delayed gastric emptying (DGE), or postoperative hemorrhage (PPH) between groups. This apparent discrepancy with the significantly increased rate of wound dehiscence is mechanistically plausible: POPF, PPH, and DGE are predominantly determined by intraoperative anatomical and technical factors, such as pancreatic gland texture, pancreatic duct diameter, the type of pancreaticoenteric anastomosis, and intrinsic patient and tumor characteristics, and are therefore largely independent of preoperative biliary drainage. In contrast, wound dehiscence is primarily driven by impaired wound healing, local inflammation, and surgical site contamination, which may in turn be promoted by the bacterial bile contamination associated with preoperative stenting. The numerical trends toward increased infectious complications and the significant increase in wound dehiscence therefore remain clinically concerning.

Hospital length of stay and ICU requirements were comparable between groups, indicating that the increased microbial burden and inflammatory response did not translate into prolonged resource utilization in our cohort. This finding is consistent with the landmark randomized trial by van der Gaag et al. [2], which similarly found no difference in hospital stay between drainage and early surgery groups, and may reflect modern perioperative care protocols and enhanced recovery pathways.

### Stent selection and timing considerations

Among patients who received preoperative stents, optimal stent type selection remains a subject of debate. Network meta-analytic evidence has demonstrated insufficient data to definitively recommend one drainage modality over another [8]. However, a study comparing self-expanding metal stents to plastic stents has suggested potential advantages of SEMS, including lower rates of cholangitis and postoperative pancreatic fistula [5]. Our study did not analyze outcomes stratified by stent type due to sample size limitations, representing an area for future investigation.

The timing of surgery following stent placement also warrants consideration. In our cohort, time to surgery was significantly longer in the stent group (51.2 vs. 23.8 days, *p* = 0.001), reflecting the interval from stent placement to definitive resection. This extended preoperative interval may contribute to the increased bacterial colonization observed, as prolonged stent dwell time has been associated with biofilm formation and ascending bacterial contamination. A recent systematic review and meta-analysis examining optimal timing after preoperative biliary drainage demonstrated that delaying surgery by 2, 3, or 4 weeks did not improve postoperative outcomes, and the authors recommended early surgery to minimize drainage-related complications [13]. A prior randomized trial found that the delay associated with preoperative drainage did not significantly impair the survival [14], suggesting that concerns about tumor progression during the drainage interval may be overstated. However, the increased complication burden observed in our study and others supports minimizing the time between stent placement and definitive surgery when drainage is deemed necessary.

In addition, post-procedural complications observed in 31.8% of stented patients in our cohort, most frequently post-ERC cholangitis (24.2%), may have contributed to the prolonged time to surgery, particularly in cases requiring antibiotic treatment or recovery from post-ERC pancreatitis prior to definitive resection.

While our findings and current evidence generally argue against routine preoperative drainage, it is important to acknowledge that specific patient subgroups may benefit from selective biliary decompression. A recent multicenter study of patients with severe obstructive jaundice (bilirubin ≥ 250 µmol/L) demonstrated that preoperative biliary drainage in this highly selected population was associated with reduced severe postoperative complications compared to direct surgery [15]. This suggests that the risk-benefit ratio of preoperative drainage may differ based on the degree of hyperbilirubinemia and patient-specific factors.

### Study implications and clinical recommendations

Our findings contribute to the growing body of evidence questioning the routine application of preoperative biliary drainage in resectable pancreatic cancer. The substantial increase in bacterial contamination, elevated postoperative inflammatory markers, and increased rate of wound complications in stented patients underscore the need for judicious patient selection when considering preoperative drainage. Recent comprehensive meta-analytic data encompassing over 33,000 patients have confirmed that preoperative biliary drainage significantly increases not only surgical site infections but also postoperative pancreatic fistula, intra-abdominal infections, and sepsis [12], reinforcing the importance of selective rather than routine drainage strategies.

Based on current evidence, preoperative biliary drainage should be reserved for specific clinical scenarios, including patients with cholangitis requiring source control, those with significant malnutrition, severe hyperbilirubinemia, or coagulopathy necessitating optimization, patients requiring neoadjuvant therapy with anticipated delays to surgery, and cases where definitive resection cannot be performed expeditiously due to logistical constraints. In the absence of these indications, proceeding directly to surgical resection appears preferable and avoids the infectious and inflammatory sequelae documented in this study.

For patients who do require preoperative drainage, strategies to minimize bacterial contamination merit consideration. These may include judicious antibiotic prophylaxis during and after stent placement, selection of SEMS over plastic stents when prolonged drainage is anticipated, and coordination between gastroenterology and surgical teams to minimize the interval between stenting and definitive surgery.

### Limitations

Several limitations of this study warrant acknowledgment. As a retrospective single-center analysis, the decision to place preoperative stents was not randomized and may have been influenced by disease severity, patient comorbidities, or referral patterns. Although baseline characteristics, bilirubin levels at presentation, and pathological tumor parameters did not differ significantly between groups, propensity score matching was not performed because of the limited sample size of the no-stent group, and residual selection bias cannot be fully excluded. The single-center design may limit generalizability to institutions with different practice patterns or patient populations.

Our analysis did not stratify outcomes by stent type or specific indications for preoperative drainage, which could provide more nuanced guidance for clinical decision-making. Long-term oncologic outcomes were not assessed in this analysis, representing an important area for future research. In the stent group, preoperative laboratory values were by definition obtained after biliary drainage and therefore reflect post-drainage rather than baseline liver function; comparability at presentation could accordingly be assessed on the basis of bilirubin only. Cholangitis at presentation was not documented as a discrete standardized variable in the retrospective records.

The relatively modest sample size, particularly in the no-stent group (*n* = 36), limited statistical power to detect differences in individual complications that occurred at low frequencies. Larger multicenter studies would provide more definitive evidence regarding the clinical impact of stent-related bacterial contamination.

## Conclusion

Preoperative biliary stenting prior to pancreatic head resection is associated with substantially increased bacterial bile contamination, prolonged operative times, and elevated postoperative inflammatory markers. While major surgical complications were not significantly increased, the heightened inflammatory response and significantly elevated rate of abdominal wound dehiscence in stented patients raise important concerns. These findings support a selective rather than routine approach to preoperative biliary drainage, with careful consideration of the infectious and inflammatory consequences when drainage is deemed necessary. This study adds a contemporary Western cohort in which the biliary microbial spectrum was predominantly polymicrobial and included Candida species in nearly one fifth of culture-positive patients. In two groups with a comparable degree of biliary obstruction at presentation, stenting was associated with wound-related rather than pancreas-specific complications. Future prospective studies examining the relationship between bacterial contamination patterns, antimicrobial strategies, and long-term outcomes would further refine clinical decision-making in this challenging patient population.

## Data Availability

The datasets generated and/or analyzed during the current study are not publicly available due to institutional data protection regulations but are available from the corresponding author on reasonable request.
